# Exploring the role of autistic traits and eating disorder psychopathology on mentalising ability in the general population

**DOI:** 10.1186/s40359-023-01306-z

**Published:** 2023-09-06

**Authors:** Kate Fithall, Indigo E Gray, Jake Linardon, Andrea Phillipou, Peter H Donaldson, Natalia Albein-Urios, Peter G Enticott, Matthew Fuller-Tyszkiewicz, Melissa Kirkovski

**Affiliations:** 1 School of Psychology, Deakin University, Geelong, Australia; 2 Centre for Youth Mental Health, The University of Melbourne, Melbourne, VIC Australia; 3 Orygen, Melbourne, VIC Australia; 4 Department of Psychological Sciences, Swinburne University of Technology, Melbourne, VIC Australia; 5 Department of Mental Health, St Vincent’s Hospital, Melbourne, VIC Australia; 6 Department of Mental Health, Austin Hospital, Melbourne, VIC Australia; 7 Institute for Health and Sport, Victoria University, Melbourne, VIC Australia

**Keywords:** Autism, Eating disorder, Mentalizing, Theory of mind, Biological sex, Female

## Abstract

**Background:**

This study evaluated the role of overlapping traits and characteristics related to autism spectrum disorder (autism) and anorexia nervosa (AN) in the general population, and the impact of these traits on mentalising ability.

**Methods:**

A sample of young adults (N = 306), aged 18–25 years, was recruited to complete an online study that consisted of 4 measures: the Autism-Spectrum Quotient, Eating Disorder Examination Questionnaire, the Mentalization Scale, and the Reading the Mind in the Eyes task.

**Results:**

Higher levels of autistic traits, particularly difficulty with attention switching, were associated with increased eating disorder psychopathology. Overall, autistic traits and eating disorder psychopathology were related among females, but not males. Difficulty with attention switching, however, was related to eating disorder psychopathology among both females and males. Autistic traits also appear to have a greater role in mentalising ability than does eating disorder psychopathology.

**Conclusion:**

The role of attention switching in overlapping traits of autism and eating disorder psychopathology needs to be more comprehensively evaluated by future research, as does the role of biological sex. Expanded knowledge in this field will help to better understand and evaluate symptoms at presentation, leading to clearer diagnoses and potentially better treatment outcomes.

## Introduction

Autism spectrum disorder (ASD; hereafter autism) and anorexia nervosa (AN) are two distinct conditions, traits of which are commonly observed to overlap [1–4]. Autism is a neurodevelopmental disorder diagnosed, on average, four times more often in males, and is characterised by marked difficulties in social communication and restrictive and repetitive behaviours and interests (RRBI: 5). In contrast, AN is a severe and life-threatening eating disorder, diagnosed more commonly in females, and is characterised by restricted eating resulting in significantly low weight, intense fear of weight gain, and distortions in body-image [5].

Despite the clinical differences, up to 30% of females seeking treatment for AN display elevated traits of, or even meet diagnostic thresholds for, autism [2, 6, 7]. This rate is significantly higher than the prevalence rate of autism in the general population, which is estimated to be around 1% [8], and perhaps emphasises the long-held view that biological, specifically female, sex might be a critical factor in the overlapping presentation of these conditions [9].

One prominent theory suggests that an altered female profile of autism, whereby RRBI could be more socially oriented than what is observed in affected males [10–12], could drive females with autism to present with RRBI around calorie intake and excessive exercise. This, therefore, likely contributes to missed or misdiagnosis among affected females, and might even precipitate the onset of an eating disorder [6]. Societal ideals of extreme thinness for women in western society, which is an etiological risk factor for AN, are one likely contributor to this [13].

Beyond this suggested atypical presentation of RRBI in autistic females, individuals with AN also display socio-cognitive impairments reminiscent of autistic symptomatology [14–17]. Females with AN, for example, have difficulty maintaining social involvement with peers despite their motivation to do so, leading them to engage in solitary activities [18], akin to the experiences of autistic females [19]. There is also growing evidence that the neurocognitive mechanisms underlying socialisation might be impaired in AN [20], again, akin to observations in autism [21].

Mentalising (or Theory of Mind; ToM) is an important aspect of social cognition that encapsulates the ability to infer and reflect on mental/emotional states of conspecifics, including their beliefs, intentions, and behaviours [22]. Individuals with AN have been shown to have reduced mentalising and empathic abilities based on both self-report [23] and performance-based behavioural measures [15]. Furthermore, there is evidence indicating that individuals with either autism or AN have overlapping neurocognitive profiles with respect to mentalising ability [24, 25]. Specifically, both groups perform similarly, and both present with more difficulty than non-clinical controls on various behavioural measures of mentalising [24, 25].

It has been suggested that mentalising difficulties could arise in AN as a result of neurocognitive effects of starvation due to highly restricted eating [26]. That is, the social-emotional and cognitive deficits, such as behavioural rigidity and social withdrawal, that are observed when the brain is deprived of nutrients may mimic what is seen in autism [27]. Indeed, there is evidence suggesting that autistic traits increase with AN symptom severity [27–29]. Importantly, there remains some controversy as to whether this relationship remains stable as AN symptoms increase or alleviate [27], or whether autistic characteristics increase and reduce together with the acute and remission stages of AN respectively [29]. Conversely, there has also been suggestion that autistic traits are more static, and present even in individuals with lesser symptom severity, persisting even when the eating disorder is in recovery [30, 31] or remission [4, 32, 33], suggesting that the overlap goes beyond factors attributable solely to the effects of under-nourishment. Furthermore, autistic traits have been argued to maintain or exacerbate symptoms of AN, leading to poorer mental health outcomes [34, 35], and thus highlighting the need to better understand this overlap.

Evidently, the role and neurocognitive impacts of starvation in AN in relation to the observed behavioural overlap with autism is poorly understood. One method by which to address this clinical confound is to evaluate the role of traits related to autism and AN on mentalising ability within a non-clinical population. Mentalising difficulty is related to sub-clinical traits of autism [36], and moreover, trait-based (i.e. self-report) measures of cognitive and effective empathy have been shown to predict sub-clinical autistic traits in the general population [37]. With respect to the role of sub-clinical eating disorder psychopathology on mentalising ability, however, the findings are mixed. For example, one study reported mentalising difficulty in adolescents at risk of developing eating disorders, based on their performance on faux pas recognition tasks, whereby participants were required to identify a social faux pas based on one agents awareness of making an inappropriate comment, and also awareness of the feelings of the second agent upon their hearing of the inappropriate comment [38], whereas no such association was identified by Bremser and Gordon [39]. No studies to date have evaluated the overlap between sub-clinical traits of autism and AN on mentalising ability.

The present study aimed to understand the overlapping role of traits and characteristics associated with autism and AN on mentalizing ability. The decision was made to target the general population to mitigate the clinical confounds described above. This approach is appropriate given the presence of such characteristics sub-clinically and in the general population. Given its critical implications in both autism and AN, the role of biological sex was also investigated. First, it was hypothesised that increased autistic traits would predict a greater presence of eating disorder psychopathology, i.e., traits related to AN. Secondly, in line with previous, albeit limited, findings, it was further expected that the relationship between traits of autism and eating disorder psychopathology would be stronger among females than males. Finally, we anticipated that individuals with higher levels of autistic traits and eating disorder psychopathology would have increased mentalising difficulty compared to those with less traits of each.

## Materials and methods

### Participants

This study received approval from the Deakin University Human Research Ethics Committee (DU-HREC: Project ID 2020-037) and was conducted in accordance with the and the Declaration of Helsinki.

Based on conservative estimates, a priori power analysis suggested that for a linear multiple regression using scores on our clinical measures as explanatory variables to predict scores on mentalising tasks (α = 0.05; power = 0.95), a total sample size of 312 participants was required (calculations conducted using G*Power [40]). A total of 329 participants completed this online study via social media (e.g., Facebook, Twitter, Reddit) and dedicated survey platforms (e.g., Prolific). Following data cleaning and quality checks, as outlined below, the final sample comprised N = 306 adults from the general population (biological sex: 217 females and 89 males, six of these participants reported being gender diverse), aged 18–25 years. This narrow age range was selected to reduce the effects of developmental confounds.

### Measures and procedure

Upon accessing the survey via Qualtrics, participants downloaded and read the plain language statement (PLS). Consent was provided via checkbox, with participants confirming that they had read and understood the PLS, and also that they were healthy adults aged between 18 and 25 years. Next, participants provided basic demographic information, and completed the measures listed below. All tasks were completed in one sitting and as none of the measures used are time-sensitive, participants were encouraged to take breaks as needed. The study was programmed so that responses could not be submitted with missing data, participants were required to have addressed every item before they could submit. Upon submission of responses, participants had the opportunity to enter a draw to win a $100AUD Amazon.com gift voucher.

#### Clinically relevant measures

Traits and characteristics of autism were measured using the Autism-Spectrum Quotient (AQ; [41]), a 50 item self-report questionnaire measured on a 4-point Likert scale specifically designed for use in non-clinical populations. In addition to a total score, the AQ also includes five subscales: social skills, attention switching, communication, imagination, and attention to detail. The AQ has good psychometric properties, including good test-retest reliability (*r* = .7) and moderate to high internal consistency across each of the five sub-scales (Communication; α = 0.65, Social; α = 0.77, Imagination; α = 0.65, Attention to Detail; α = 0.63, Attention Switching; α = 0.67) [41]. For the purpose of this manuscript, the AQ was scored according to the original (binary) protocol [41]. High scores on the AQ are reflective of an increased presence of autistic traits and characteristics.

Characteristics of AN were assessed using the Eating Disorder Examination Questionnaire (EDE-Q; 42). This 28 item self-report measure was developed based on the Eating Disorder Examination Semi-structured Interview [43], is suitable for use in both clinical and non-clinical populations, and includes four subscales: restraint, eating concern, body shape concern and weight concern. The EDE-Q is suitable for non-clinical populations, and has sound psychometric properties; test-retest reliability (*r* = .66 to 0.94) and internal consistency (α = 0.70 to 0.93) [44]. Higher scores on the EDE-Q are indicative of increased eating disorder psychopathology.

#### Mentalising measures

The Mentalization Scale (MentS; [45]) is a 28 item self-report measure of mentalising ability/insight which is completed on a 5-point Likert scale and is suitable for non-clinical use. The MentS provides a total score indicative of self-reported mentalising ability, as well as sub-scales related to the Self, Others, and Motivation. This measure has good internal consistency (α = 0.84), and higher scores on the MentS are indicative of increased mentalization ability [45].

Participants also completed a computerised version of the Reading the Mind in the Eyes task (RMET; 46). This behavioural task requires participants to view 36 photographs of an actor’s eyes, presented in the centre of the screen one at a time. Participants were asked to select from four provided options, the response that best described the actor’s emotional state. Average response accuracy was calculated. This task is widely used and forms part of the “Social” component of the National Institute of Health (NIH) Research Domain Criteria (RDoC) framework [47].

#### Data quality assessment and cleaning

A conservative approach was taken towards data cleaning. First, IP addresses and Prolific IDs were screened and when multiple completions from the same source were identified, all were excluded. Response sets were then excluded if the reported age violated the age criterion. Submissions completed in less than 10 minutes were excluded as this time was deemed insufficient to properly complete the tasks, and finally, incomplete submissions were also removed.

Data were then removed if the responses to open-ended questions were nonsensical (i.e., not words, or not likely completed by a human) or not in English. Datasets were also excluded if patterned or repetitive selection of response options was present.

Some demographic datapoints (i.e., height and weight), were also removed due to input errors (e.g., unclear, or ambiguous responses such as unclear metrics). While participants were instructed to use a metric scale (i.e., centimetres and kilograms respectively), many did not. When imperial measurements were used and explicitly stated, these were converted into metric measurements. When the scale was not clear, those data points were excluded.

#### Statistical analyses

Data were analysed in IBM SPSS version 28. Given the online nature of data collection, as an additional measure to ensure data quality, internal consistency (Cronbach’s alpha) was calculated for each of the subscales of the self-report questionnaires. Data were screened for statistical outliers, and outliers were windsorized. Data distributions were then inspected. Data were roughly normal, and skewness and kurtosis values for all tested variables fell within the acceptable range (i.e., between +/- 2 and 7 respectively [48]). All relevant statistical assumptions of the general linear model (i.e., linearity, normality, independence, homoscedasticity and multicollinearity) were assessed and met. Behavioural/clinical profiles were summarised to characterise the sample. Linear multiple regression analyses were performed to test the hypothesis that increased autistic traits would predict a greater presence of eating disorder psychopathology. Correlation analyses were conducted to assess the relationship between traits of autism and eating disorder psychopathology in females and males, and the strength of these relationships were compared using Fisher’s z tests. Finally, a series of one-way analyses of variance (ANOVAs) were conducted to assess whether participants with various combination of high/low levels of traits related to autism and eating disorder psychopathology differed on mentalising outcomes.

## Results

### Reliability index

Within the present sample, moderate to high internal consistency was noted across each of the five sub-scales of the AQ (communication; α = 0.74, social skills; α = 0.82, imagination; α = 0.56, attention to detail; α = 0.66, and attention switching; α = 0.71), the four sub-scales of the EDE-Q (restraint; α = 0.83, eating concern; α = 0.83, shape concern; α = 0.88, and weight concern; α = 0.81), and the three sub-scales of the MentS (self; α = 0.81, other; α = 810, and motivation; α = 68).

### Summary of behavioural/clinical profiles

Sample demographics are presented in Table 1, and a summary of scores and descriptive statistics for each of the measures and their subscales are provided in Table 2. Due to the discrepancy in sample sizes when data were stratified by sex, Mann-Whitney U tests were conducted to determine whether there was a difference in scores on each measure between females and males. Females scored lower than males on the imagination subscale of the AQ, and scored higher than males on all subscales of the EDE-Q. Regarding mentalizing outcomes, females scored higher than males on the other and motivation subscales of the Ments, and also on RMET accuracy.

**Table 1:** Summary of participant demographics

		*n*	Mean (SD)	Range	Median
Age (years)	Female	217	22.09(2.04)	18–25	22.00
	Male	89	22.09(2.14)	18–25	22.00
	Total	306	22.09(2.07)	18–25	22.00
Height (cm) ^a^	Female	203	163.60(7.93)	117–186	164.00
	Male	85	176.19(7.08)	153–191	177.00
	Total	288	167.31(9.60)	117–191	167.00
Weight (kg) ^a^	Female	203	63.95(18.48)	40–155	59.00
	Male	82	76.13(14.52)	49–130	75.00
	Total	285	67.46(18.26)	40–155	63.50
BMI ^a^	Female	195	23.89(6.56)	14–61	21.91
	Male	81	24.32(4.08)	16–39	23.62
	Total	276	24.01(5.94)	14–61	22.62

Note: BMI = body mass index. ^a^ missing data due to ambiguity in responses/metrics.

**Table 2:** Summary of scores on self-report and behavioural measures

		Mean (SD)	Median	U	*p*
AQ					
Total	Female	18.57(7.80)	18.00		
	Male	19.42(7.49)	20.00	8952.0	0.316
	Total	18.80(7.66)	18.00		
Social Skills	Female	3.00(2.53)	2.00		
	Male	2.87(2.61)	2.00	9259.0	0.568
	Total	2.96(2.55)	2.00		
Attention Switching	Female	5.02(2.38)	5.00		
	Male	4.89(2.25)	5.00	9505.5	0.828
	Total	4.98(2.34)	5.00		
Attention to Detail	Female	5.05(2.18)	5.00		
	Male	5.33(2.13)	5.00	9025.0	0.364
	Total	5.13(2.16)	5.00		
Communication	Female	2.89(2.36)	3.00		
	Male	3.30(2.17)	3.00	8444.5	0.082
	Total	3.01(2.31)	3.00		
Imagination	Female	2.60(1.77)	2.00		
	Male	3.03(1.76)	3.00	8208.0	0.036*
	Total	2.73(1.78)	3.00		
EDE-Q					
Global	Female	1.90(1.40)	1.75		
	Male	1.44(1.22)	1.05	7909.0	0.013*
	Total	1.77(1.37)	1.40		
Restraint	Female	1.56 (1.60)	1.00		
	Male	1.13 (1.26)	0.80	8193.5	0.036*
	Total	1.43 (1.52)	1.00		
Eating Concern	Female	1.25 (1.37)	0.80		
	Male	0.90 (1.25)	0.20	7906.0	0.012*
	Total	1.15 (1.35)	0.60		
Shape Concern	Female	2.42 (1.57)	2.44		
	Male	1.90 (1.42)	1.56	7852.5	0.010*
	Total	2.27 (1.54)	2.11		
Weight Concern	Female	2.38 (1.75)	2.20		
	Male	1.85 (1.60)	1.40	8037.0	0.021*
	Total	2.23 (1.72)	1.80		
MentS					
Total	Female	102.11(14.66)	101.00		
	Male	98.96(12.58)	99.00	8373.5	0.068
	Total	101.19(14.14)	100.00		
Self	Female	22.74 (7.93)	22.00		
	Male	23.43 (6.87)	24.00	9206.0	0.521
	Total	22.94 (7.63)	23.00		
Other	Female	39.70 (5.57)	40.00		
	Male	38.18 (5.04)	39.00	7833.5	0.009*
	Total	39.25 (5.45)	40.00		
Motivation	Female	39.67 (5.12)	40.00		
	Male	37.35 (5.43)	38.00	7320.0	< 0.001*
	Total	39.00 (5.31)	39.00		
RMET					
	Female	25.83(4.17)	26.00		
	Male	24.62(4.40)	25.00	8187.5	0.036
	Total	25.48(4.27)	26.00		

*Note.* AQ = Autism spectrum quotient, EDE-Q = Eating Disorder Examination Questionnaire, MentS = The Mentalization Scale, RMET = Reading the Mind in the Eyes task. * statistically significant, α = 0.05

### Autistic traits as a predictor of eating disorder behaviour

A linear regression indicated that autistic traits (AQ total score) were significantly associated with eating disorder psychopathology (EDE-Q global score), *β =* 0.268, *t* = 4.857, *p* < .001. Autistic traits explained 7% of the variance, *R*^*2*^ = 0.07, *F*(1, 304) = 23.94, *p* < .001. To explore which, if any, specific traits of autism explained the most variance, a second linear regression that included all AQ sub-scales was conducted. This identified that the attention switching subscale of the AQ significantly predicted global EDE-Q score *β =* 0.204, *t* = 3.029, *p* = .003. Table 3 details regression coefficients for AQ sub-scales and Pearson correlations between autistic traits and eating disorder psychopathology.

**Table 3:** Regression Coefficients for Predicting Eating Disorder Behaviour (EDE-Q) based on AQ subscales

AQ sub-scale	Pearson Correlation			Linear Regression				
	*r*	*p*		B	95% CI	β	*t*	*p*
Social Skills	0.22	< 0.001		0.017	[-0.07, 0.10]	0.031	0.394	0.694
Attention Switching	0.28	< 0.001		0.119	[0.04, 0.20]	0.204	3.029	0.003*
Attention to Detail	0.05	0.360		0.001	[-0.07, 0.07]	0.002	0.040	0.968
Communication	0.23	< 0.001		0.063	[-0.03, 0.16]	0.107	1.350	0.178
Imagination	13	0.029		0.004	[-0.09, 0.10]	0.005	0.073	0.941

*Note.* CI confidence interval for *B.* AQ = Autism spectrum quotient. * statistically significant, α = 0.05

### The role of biological sex

To investigate the role of biological sex, data were stratified and two independent correlation analyses were conducted due to the relatively small sample size of male cohort limiting our ability to conduct a regression for that group. There was a moderate significant relationship between autistic traits (AQ) and eating disorder psychopathology (EDE-Q) for the female, *r* (215) *=* 0.314, *p* < .001, but not the male, *r* (87) *=* 0.180, *p =* .091 cohort. Fisher’s z-test, however, revealed no significant difference between the strength of these correlations; *z* = − 1.09, *p* = .14. As Pearson’s correlations are not very robust to violations of statistical assumptions, sensitivity analyses were conducted using Spearman’s rank-order correlations as the EDE-Q distribution appeared to be visually skewed, despite acceptable skewness values (absolute skew = 0.73). These analyses showed a similar pattern of results whereby there was a significant relationship between AQ and EDE-Q scores for *r* (215) *=* 0.294, *p* < .001, but not males *p =* .089.

Due to the attention switching subscale of the AQ being identified as a significant predictor variable of eating disorder psychopathology, this too was evaluated by biological sex. Significant correlations were found between attention switching and EDE-Q global scores for the female *r* (215) *=* 0.294, *p* < .001 and male cohorts *r* (87) *=* 0.224, *p =* .035, but again, Fisher’s z-test again revealed no significant difference between the strength of these correlations, *z* = − 0.59, *p* = .28. Sensitivity analyses using Spearman’s rank-order correlations again supported the findings of the primary analyses, whereby there was a significant relationship between these variables among females *r* (215) *=* 0.251, *p* < .001 and males *r* (87) *=* 0.228, *p* < .035.

### The role of autistic traits and characteristics of AN on mentalising ability

Pearson correlations (two-tailed) revealed significant, albeit weak, negative relationships between the MentS total score and AQ, *r* (304) = − 0.36, *p* < .001. A non-significant trend was identified between the MentS total score and the EDE-Q, *r* (304) = − 0.12, *p* = .037). Correlation analyses also revealed that the RMET accuracy and AQ scores were negatively correlated, *r*(304) = − 0.15, *p* = .010, however, the RMET and EDE-Q were not, *r*(304) = − 0.06, *p* = .268. To account for multiple comparisons, alpha was set at *p* < .01 based on a Bonferroni correction.

A linear multiple regression was conducted to determine whether individuals with greater levels of traits associated with autism and eating disorder psychopathology would have increased self-reported mentalising difficulty (as measured via the MentS-Total). Results indicated that 13% of the variance in mentalising ability was accounted for by autistic traits and eating disorder psychopathology (*R*^*2*^ = 0.13, *F*(2, 303) = 22.33, *p* < .001). Autistic traits significantly predicted mentalising ability (*β* = − 0.35, *p* < .001), however, AN traits did not (*β* = − 0.03, *p* = .65).

A second linear multiple regression was conducted to determine whether individuals with more traits associated with autism and eating disorder psychopathology would have increased difficulty in accurately completing a behavioural mentalising task (the RMET). These traits explained 2% of the variance (*R*^*2*^ = 0.02, *F*(2, 303) = 3.41, *p* < .05). Autistic traits significantly predicted mentalising ability (*β* = − 0.14, *p* = .02), however, traits of AN did not (*β* = − 0.03, *p* = .66).

To explore this further, data were split into quadrants based on their combination of high/low levels of traits related to autism and eating disorder psychopathology, as determined by a median split for each measure (i.e., the AQ and EDE-Q respectively). Scores on both mentalising measures were then compared between the four groups. RMET accuracy did not differ between groups based on their combination of traits related to autism or eating disorder psychopathology. Significant differences between these groups were, however, identified for scores on the MentS and each of its sub-scales. Refer to Table 4 for descriptive statistics and results of one-way analysis of variance (ANOVA). Tukey’s post-hoc analyses are presented in Table 5. Group differences appear between those with high vs. low levels of autistic traits, seemingly irrespective of the level of eating disorder psychopathology present.

**Table 4:** Descriptive statistics and one-way ANOVA results comparing mentalising outcomes based on combinations autistic traits and eating disorder psychopathology

	High AQ – High EDE-Q	High AQ – Low EDE-Q	Low AQ – High EDE-Q	Low AQ – Low EDE-Q	F (3,302)	*p*	η2
	n = 93	n = 66	n = 60	n = 87			
	M (SD)						
**RMET**							
Accuracy	24.55 (4.91)	25.62 (4.14)	26.02 (4.01)	25.99 (3.66)	2.26	0.082	0.02
**MentS**							
Total	96.60 (13.99)	96.00 (13.50)	104.43 (12.54)	107.79 (12.61)	15.56	< 0.001*	0.13
Self	19.44 (7.48)	21.74 (6.95)	24.48 (7.09)	26.52 (6.84)	16.41	< 0.001*	0.14
Other	38.68 (5.43)	36.55 (5.69)	40.30 (5.10)	41.21 (4.58)	11.26	< 0.001*	0.10
Motivation	38.48 (5.36)	37.71 (5.52)	39.65 (4.85)	40.07 (5.20)	3.13	0.026*	0.03

Note: * statistically significant, α = 0.05

**Table 5:** Results of Tukey’s post-hoc analysis

	Group		*p*	99% Confidence Interval	
				Lower Bound	Upper Bound
MentSTotal	High AQ - High EDE-Q	High AQ - Low EDE-Q	0.992	-6.08	7.28
		Low AQ - High EDE-Q	0.002*	-14.70	-0.96
		Low AQ - Low EDE-Q	< 0.001*	-17.38	-5.00
	High AQ - Low EDE-Q	Low AQ - High EDE-Q	0.002*	-15.84	-1.03
		Low AQ - Low EDE-Q	< 0.001*	-18.57	-5.02
	Low AQ - High EDE-Q	Low AQ - Low EDE-Q	0.43	-10.33	3.61
MentS	High AQ - High EDE-Q	High AQ - Low EDE-Q	0.186	-5.89	1.29
		Low AQ - High EDE-Q	< 0.001*	-8.74	-1.35
		Low AQ - Low EDE-Q	< 0.001*	-10.41	-3.75
	High AQ - Low EDE-Q	Low AQ - High EDE-Q	0.137	-6.72	1.24
		Low AQ - Low EDE-Q	< 0.001*	-8.42	-1.13
	Low AQ - High EDE-Q	Low AQ - Low EDE-Q	0.323	-5.78	1.71
MentO	High AQ - High EDE-Q	High AQ - Low EDE-Q	0.055	-0.49	4.76
		Low AQ - High EDE-Q	0.237	-4.33	1.08
		Low AQ - Low EDE-Q	0.007*	-4.96	-0.1
	High AQ - Low EDE-Q	Low AQ - High EDE-Q	< 0.001*	-6.67	-0.84
		Low AQ - Low EDE-Q	< 0.001*	-7.33	-2.00
	Low AQ - High EDE-Q	Low AQ - Low EDE-Q	0.726	-3.65	1.83
MentM	High AQ - High EDE-Q	High AQ - Low EDE-Q	0.798	-1.88	3.43
		Low AQ - High EDE-Q	0.538	-3.90	1.56
		Low AQ - Low EDE-Q	0.182	-4.04	0.87
	High AQ - Low EDE-Q	Low AQ - High EDE-Q	0.166	-4.88	1.00
		Low AQ - Low EDE-Q	0.032*	-5.05	0.34
	Low AQ - High EDE-Q	Low AQ - Low EDE-Q	0.964	-3.19	2.35

Note: * statistically significant, α = 0.05

## Discussion

The present study sought to investigate the relationship between traits associated with autism and eating disorder psychopathology in a non-clinical young adult population recruited from the general population. The role of overlapping traits and characteristics associated with autism and eating disorder psychopathology on mentalising ability was also investigated. As expected, higher levels of autistic traits were associated with higher levels of eating disorder psychopathology. When data were stratified by sex, a significant positive relationship was identified between traits of autism and eating disorder psychopathology among females, but not males, though attention switching was related to eating disorder psychopathology in both groups. The strength of these correlations, however, did not differ between males and females. Finally, the hypothesis that traits of autism and eating disorder psychopathology would impact upon mentalising ability was partially supported, as only autistic traits were noted to significantly account for variance in indexes of mentalising abilities. Further, when data were grouped based on combinations of high versus low traits of autism and eating disorder psychopathology, differences in mentalising were only present between those with high and low levels of autistic traits, irrespective of the level of traits related to AN present.

### The association between autistic traits and eating disorder psychopathology

That autistic traits were positively associated with eating disorder psychopathology is in line with previous studies in clinical [1] and non-clinical samples [49, 50], providing further evidence for overlapping profiles of autism and AN that extend beyond diagnostic features or acute effects of starvation [51]. Indeed, autistic traits have been proposed as a risk factor for the development of AN, with some arguing that a shared underlying genetic vulnerability exists [9, 52]. One study, for example, reported that 10% of female adolescents with AN met criteria for autism based on current symptoms and developmental history obtained through a validated parental report measure [28], suggesting that autistic traits were present in females with AN even prior to their presentation at eating disorder services. This is in line with longitudinal research suggesting that increased autistic-like social traits in childhood precede disordered eating behaviours in adolescence [53].

Exploring this further, we identified that attention switching was the only AQ-subscale that contributed to eating disorder psychopathology. This finding is in line with two previous studies evaluating eating disorder psychopathology and autistic traits in the general population that similarly implicate only the attention switching subscale of the AQ in eating disorder psychopathology [54, 55]. Difficulty in switching, or shifting, attention or focus is a common difficulty experienced by individuals with autism and also by those with AN [56–61], and difficulty in this domain can manifest cognitively (i.e., rigid thinking) and behaviourally (i.e., inflexibility, stereotyped behaviour) [62]. Such difficulties in executive function likely contribute to the presence of RRBI in autism [63, 64] and also to rigid beliefs about weight and restricted eating behaviours in AN [65]. Furthermore, these rigid beliefs and behaviours in AN have themselves been associated with the RRBI domain of autism [6]. This finding therefore supports the notion that RRBI are likely an important contributing factor to the overlap between autism and AN.

Unlike autistic traits overall, which were only related to eating disorder psychopathology for the female sub-sample, difficulty with attention switching was related to increased eating disorder psychopathology in both females and males. In comparison to the few studies that have evaluated the role of biological sex in this domain, the relationship between autistic traits and eating disorder psychopathology has previously been reported as being stronger among females [49, 66], a finding which was not replicated by the present study. Taking these findings together, it is logical to suggest that even sub-clinically, autistic traits contribute to the presence of eating disorder psychopathology, and that this might be more salient among females [53, 67]. Indeed, among our sample, females reported significantly higher levels of eating disorder psychopathology than males. Given the critical role of biological sex in both autism [11] and AN [68] clinically, this is in an area in need of further investigation.

### The role of autistic traits and eating disorder psychopathology on mentalising ability

With respect to mentalising, eating disorder psychopathology was negatively correlated with self-reported mentalising ability, but not related to accuracy on the behavioural task. This finding is in line with the broader literature which reports a discrepancy between self-report and behavioural performance [50, 60]. Specifically, an association between non-clinical eating disorder psychopathology and poorer mentalising ability based on a self-report measure was established in one study [69], but not in others where a behavioural mentalising task (specifically, the RMET) was used [38, 39]. A similar conclusion was reached by Donaldson and colleagues [37] with respect to the ability of state- versus trait- based measures of cognitive and effective empathy to predict sub-clinical autistic traits. Together, these finding highlight a discrepancy in self-reported versus behavioural mentalising measure which warrants further investigation. A meta-analysis conducted by Murphy and Lilienfeld [70] attempt to clarify this, reporting that individuals may not provide valid appraisals of their own mentalising abilities based on comparisons between behavioural and self-report measures. Participants may therefore have rated their abilities inaccurately by inflating their perceived mentalising skills. Nonetheless, the MentS and RMET are validated measures of mentalising ability, and further understanding of the processes underlying the overlap of autistic and eating disorder traits is justified.

Finally, mentalising was predominantly affected by autistic traits. While the combination of elevated autistic traits and eating disorder psychopathology together predicted mentalising ability within this sample, only AQ scores were found to be statistically significant. Moreover, stratifying data into quadrants based on combinations of high/low levels of autistic traits and eating disorder psychopathology suggested that group differences in mentalising ability were mostly present between groups based on autistic traits, irrespective of the level of eating disorder psychopathology present. The predominately female participant sample in the present dataset might provide some explanation for this outcome. While research on the role of biological sex in autism is limited, there is some evidence that females [71], including those with autism [72], might have less difficulty in the social domain than males, though it is important to consider the role of camouflaging [73]. At a neurobiological level autistic females might even perform similarly to neurotypical males on such tasks [74, 75]. In terms of AN, one study found no quantitative differences between females with AN, with AN and elevated autistic traits, or matched controls on a behavioural measure of mentalising [76]. It is possible, therefore, that females with sub-threshold autistic and/or eating disorder psychopathology may also have less difficulty mentalising, though further research is needed to better understand the role of biological sex in this regard. Stemming from this, another critical consideration here is the possibility that the present results are reflective of less difficulty due to the assessment of a non-clinical cohort. That is, the influence of eating disorder psychopathology and autistic traits on mentalising ability is not as strong as what would be observed with higher, i.e., clinically significant symptomatology. Our findings suggest that at the non-clinical level, among the general population, no clear association exists between eating disorder psychopathology and mentalising ability. It is possible that if characteristics of AN progressed to become clinically significant, deficits may be more pronounced [27–29, 77]. In the present study, less than 10% of participants scored in the clinical range (i.e., > 4) on the EDE-Q.

### Limitations

There are some limitations of the present study to be acknowledged. The comparatively smaller male sub-sample in our study impacted our ability to thoroughly examine the role of biological sex, an area in critical need of deeper investigation. Additionally, the present sample did not allow for comparisons to be made based on both biological sex *and* gender identity. As too few respondents reported being gender diverse, statistical comparisons were impossible. There were also some methodological limitations to note. First, we acknowledge that the clinically relevant measures used relied on self-report, which may have impacted the way participants represent themselves, as well as their own level of insight. Regarding the EDE-Q specifically, it should be noted that this measure encapsulates eating disorder psychopathology broadly, rather than a specific focus on AN. Clinically, there is strongest evidence for a link between autism and AN [78], however in a study of the general population, as presented here, the research investigating the relationship between autistic traits and broader eating disorder psychopathology is well documented [50], and hence this measure is appropriate in this context. Given the online nature of the study, we were unable to conduct formal clinical and cognitive assessments, the latter being an important consideration as intelligence has some relevance to performance on the RMET [79]. Furthermore, while some participants exceeded clinical “cut-offs”, in the absence of formal assessment, it cannot be determined for certain whether clinically significant symptomatology was present. As all measures used here are considered appropriate for use in the general population, however, we are confident in our outcomes. Further to this point, we could not be certain about participants’ mental-health history. While mental health history is an important consideration for future research in this, or any other field related to mental health, one study reported that a correlation between eating disorder psychopathology and autistic traits exists independent of traits related to anxiety and depression [49]. Another found that this was only true for female participants [66].

### Direction for future research

While the present study was specifically designed to assess the role of sub-clinical traits of autism and eating disorder psychopathology on mentalising ability, our results indicate that future work should consider both elements of the autistic dyad, i.e., social difficulty and RRBI, when investigating the overlap between autism and AN/eating disorder psychopathology, both clinically and sub-clinically. Furthermore, considering the knowledge that the male and female profiles of autism might be clinically different [80, 81], future research should incorporate measures developed specifically to capture the female profile of autism. The modified version of the Girls Questionnaire for Autism Spectrum Condition [78] is one example of such a measure. Finally, as rates of gender diversity are higher among neurodiverse cohorts [82, 83], and eating disorder risk is higher among gender diverse communities [84–86], it is important that both biological sex *and* gender identity are considered in future research.

### Summary and conclusion

We provide some insight into the overlapping nature of autistic traits and eating disorder psychopathology in a young adult sample of the general population. In this population, eating disorder psychopathology appears to have little impact on mentalising ability. RRBI, possibly as a consequence of set-shifting deficits, may be an important factor in understanding the overlap in features related to autism and AN. Future research should assess the autism dyad more comprehensively, and also further consider the role of biological sex. Improved knowledge in this field will serve to develop risk evaluation for such clinical diagnoses as autism or AN, and might also contribute to improving intervention strategies tailored to individual needs.

## Data Availability

The datasets used and/or analysed during the current study are available from the corresponding author on reasonable request.
